# Liver X Receptor Expression and Pentraxin 3 Production in Chronic Rhinosinusitis and Sinonasal Mucosal Fibroblast Cells

**DOI:** 10.3390/jcm10030452

**Published:** 2021-01-25

**Authors:** Yih-Jeng Tsai, Ping-Hung Shen, Sheng-Dean Luo, Wen-Bin Wu

**Affiliations:** 1 Department of Otolaryngology Head and Neck Surgery, Shin Kong Wu Ho-Su Memorial Hospital, Taipei 11101, Taiwan; tigertsai@msn.com; 2 School of Medicine, Fu Jen Catholic University, New Taipei City 242062, Taiwan; 3 Department of Otolaryngology, Kuang-Tien General Hospital, Taichung 43303, Taiwan; allentube211@gmail.com; 4 Department of Nursing, Hung-Kuang University, Taichung 433304, Taiwan; 5 Department of Otolaryngology, Kaohsiung Chang Gung Memorial Hospital and Chang Gung University College of Medicine, Kaohsiung 833253, Taiwan; rsd0323.tw@gmail.com; 6 Graduate Institute of Clinical Medical Sciences, College of Medicine, Chang Gung University, Taoyuan 33302, Taiwan; 7 Graduate Institute of Biomedical and Pharmaceutical Science, Fu Jen Catholic University, New Taipei City 242062, Taiwan

**Keywords:** GW3965, LXR, nuclear receptor, pentraxin, PTX3, PI3K/Akt

## Abstract

The long pentraxin 3 (PTX3) is a prototypic molecule for recognizing pathogens. Liver X receptors (LXRs), belonging to nuclear receptors (NRs) for cholesterol metabolism through heterodimerizing with other NRs, were recently reported to participate in inflammation. However, their roles in chronic rhinosinusitis without nasal polyps (CRSsNP) are unclear. Therefore, this study was sought to explore roles of LXRs in chronic rhinosinusitis (CRS) sinonasal tissues and derived fibroblasts. Immunohistochemistry indicated that LXRα and β expression and lipid/fat deposition were differentially expressed in the control and CRSsNP nasal mucosa. GW7647 (a peroxisome proliferator activated receptor α (PPARα) agonist) and GW3965 (a dual agonist for LXRα and β) significantly caused PTX3 induction in the fibroblast cells. GW3965 induced PTX3 mRNA and protein expression, and the induction substantially led to PTX3 secretion. Meanwhile, an endogenous agonist-cholesterol had a similar enhancing effect on the induction of PTX3 protein. LXR siRNA knockdown to lower LXRα or β expression significantly compromised PTX3 induction. Interestingly, GW3965 also induced phosphoinositide 3-kinase/protein kinase B (PI3K/Akt) activation and its inhibition reduced PTX3 expression. Collectively, we demonstrated here for the first time that CRSsNP nasal mucosa differentially expresses LXRα and β and deposits lipids/fats that may contain cholesterol metabolites to activate LXRs. Activation of LXRs leads to PTX3 production in sinonasal mucosa-derived fibroblasts. Our previous study showed PTX3 overexpression in the nasal cavity of CRSsNP, whereas this study highlights that cholesterol metabolites and LXR activation regulate PTX3 production and may contribute to antimicrobial activity and tissue repair during CRSsNP progression.

## 1. Introduction

Nuclear receptors (NRs) such as peroxisome proliferator-activated receptor γ (PPARγ) and liver X receptor (LXR) play critical roles in lipid metabolism and inflammation [1]. The endogenous agonists for PPARγ [2] are fatty acids, whereas those for LXR are oxysterols [3].

LXRs were originally discovered as orphan receptors without corresponding ligands but were subsequently identified as NRs for an oxidation metabolite of cholesterol, namely oxysterols. LXRs consist of two different isoforms (isotypes), including LXRα and β. They share large sequence homology but with distinct tissue distributions [4]. Among them, LXRα is highly expressed in the small intestine, liver and kidney with a high degree of expression in adipose tissue and macrophages, but only a little in other tissues. In contrast, LXRβ is expressed in whole body tissue cells [5].

LXRα and β, two different isoforms, can heterodimerize with retinoid X receptor (RXR) [6,7]. Therefore, the LXR/RXR dimer can be triggered by the LXR or RXR agent. RXR belongs to the receptor of retinoic acid (RA). If LXR and RXR can be simultaneously activated, they can trigger greater activation. At present, the most common one is that the LXR/RXR dimer will bind to a sequence located at DNA. This specific sequence is called an LXR response element (LXRE) [8]. To update, LXRs are known to be related to physiological functions including de novo synthesis [9,10,11], secretion and detoxification of cholic acid and lipids [12,13,14], blood glucose homeostasis [15], immunity and infection [11,16], skin generation [17] and neuron-related functions [18].

Chronic rhinosinusitis (CRS), generally subclassified into with or without nasal polyps (CRSwNP and CRSsNP) [19], is an inflammatory process in paranasal sinuses that occurs over more than 12 weeks [20,21]. The pathogenesis and progression of CRS remains not very clear, but bacteria are believed to play a certain role in CRS propagation [22]. It is plausible, in CRS, that mucociliary clearance and host defense ability are impaired so that the sinus cavity loses its normal sterility and is colonized by the nasal flora [23]. Several studies have demonstrated that one of the pathogens, *Staphylococcus aureus*, plays a role in CRS pathogenesis. Its existence in the foci can also affect how recurrent CRS is treated [24,25]. In this regard, recognition of the pathogen and its associated molecular patterns in initiating immune response relies on pattern recognition molecules (PRMs) [26].

Pentraxin 3 (PTX3) is a prototypic PRM for microbial recognition [27], which can be produced in the myeloid cell lineage, fibroblasts, adipocytes, astrocytes and vascular, epithelial, mesangial and microglial cells, and is involved in engagement of TLR by lipopolysaccharide (LPS) and peptidoglycan [28], and proinflammatory stimuli by TNF-α and IL-1β [29,30]. Our lab also found that overproduction of PTX3 in nasal specimens and secretions of CRSsNP, but not CRSwNP [28]. Recently, PTX3 was reported to participate in tissue repair and remodeling [31,32] and in regulating complement-dependent inflammation in cancer [33].

A previous study showed that cholesterol deposits in the paranasal sinus [34]. Given that fatty acids and cholesterol metabolites are endogenous ligands for the NRs, such as PPARs and LXRs, and both of them may have a role in inflammation, this study focused on how NR agonists affect PTX3 production in primary cultured human fibroblasts obtained from CRSsNP nasal mucosa, namely human nasal mucosa-derived fibroblasts (hNMDFs). Screening revealed that GW7647 (a PPARα agonist) and GW3965 (an inducer for LXRα and β), were found to cause PTX3 expression in the hNMDFs. The LXRs and fat/lipid distribution in CRSsNP nasal mucosa tissues and possible mechanism of LXR activation on PTX3 expression were investigated in this study.

## 2. Experimental Section

Materials: Lutein (dissolved in DMSO) and lycopene (in tetrahydrofuran) were from Extrasynthese (Genay Cedex, France) and β-carotene was from Nacalai Tesque, Inc. (Kyoto, Japan). Water-soluble cholesterol, all-trans retinoic acid (RA), rosiglitazone, and retinol were from Sigma-Aldrich Chemical Co. (St Louis, MO, USA). RAR/RXR agonists/antagonists were from Tocris Cookson Ltd. (Bristol, BS, UK). The antibodies (Abs) for PI3K and Akt were purchased from Cell Signaling Technology, Inc. (Danvers, MA, USA). The Ab for PTX3 was from Abcam (Cambridge, MA, USA), whereas the Ab raised against α-tubulin (GTX628802) was purchased from GeneTex (Irvine, CA, USA).

Patient recruitment: Ten CRSsNP patients and eight controls were recruited. This study was approved by the IRB Committee (The Shin Kong Wu Ho-Su Memorial Hospital, Taipei, Taiwan; ethical approval number: 20151211R). Participants enrolled in this study were provided with informed consent before study entry. The diagnosis of CRSsNP and preparation of mucosae were described previously [28]. Briefly, CRSsNP was diagnosed based on patient history and the findings from anterior rhinoscopy, nasal endoscopy and sinus computed tomography. None of the patients had a history of allergy, asthma or aspirin sensitivity, and none had been treated with oral or topical antiallergic agent or steroid for at least two months, and none of these cases were in an actively infected status. The antibiotics were not given before the surgical procedure. The nasal mucosa was obtained through the procedure of uncinectomy and ethmoidectomy during functional endoscopic sinus surgery (FESS). The ethmoidal mucosae and the mucosae around the osteomeatal complex were collected. In the control group, there were patients with blockage in the lacrimal drainage system and were free of other nasal diseases. The agger nasi sinus cell mucosae were prepared during dacryocystorhinotomy procedures.

Immunocytochemistry (IHC) of LXR expression and lipid/fat deposition in nasal mucosa: For LXR staining, the immunocytochemistry (IHC) procedure of protein expression in nasal mucosa tissues was previously described by our lab [28]. For measuring lipid deposition, tissues were incubated with 0.05% Oil Red O in polyethylene glycol for 15 min at room temperature and then stained with hematoxylin for 30 s, washed and finally mounted with 100% glycerol.

Cell cultures: Primary cultured fibroblasts were prepared from CRSsNP nasal tissues. In short, a fragment of nasal tissue was placed in a culture dish in DMEM containing FBS (10%) and antibiotic and antimycotic (penicillin, streptomycin, and amphotericin B) (Thermo Fisher) at 37 °C in a CO_2_ incubator. The migrating cells were fibroblast cells and had been characterized [35].

Real-time PCR and RT-PCR analysis of mRNA expression: The real-time and RT-PCR were performed with the primers sets (Table 1). The PCRs were performed in a Real-Time PCR System (Life Technologies, Applied Biosystems, NY, USA). The RT-PCR was performed as previously reported [36].

Western blot analysis: Protein expression and phosphorylation were analyzed by western blotting as previously described [37].

Measurement of PTX3 secretion: The ELISA development kit for PTX3 (R&D Systems, Inc., MN, USA) was used to measure the PTX3 expression level in culture medium. The PTX3 level was measured at 450 nm and the absolute concentrations of PTX3 was obtained based on the absorbance of each standard.

SiRNA interference: Gene-specific LXR siRNAs or nonspecific control was transfected into cells using DharmaFECT according to the supplier’s instructions (Thermo Fisher Scientific). To assess knockdown efficiency and PTX3 expression, transfected cells were collected for subsequent analysis by Western blotting.

Statistical analysis: The data were presented as the mean ± standard error mean (SEM). The statistical differences between control and experimental groups were evaluated using the unpaired two-tailed Student’s *t* test. * *p* < 0.05, ** *p* < 0.01, and *** *p* < 0.001 indicate significant.

## 3. Results

### 3.1. LXRα and β Expression and Lipid/Fat Deposition in CRSsNP Nasal Mucosae

**Figure 1:**
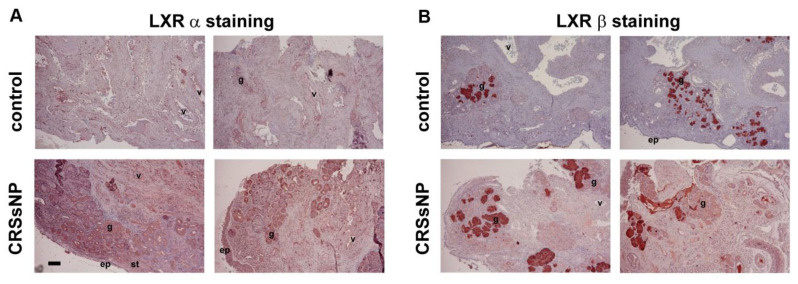
LXR distribution in human nasal mucosa. The (**A**) LXRα and (**B**) LXRβ distributions in respective nasal mucosae were determined by immunocytochemistry (IHC). Scale bar = 100 μm. LXR: Liver X receptor.

The expression of LXR has not been investigated in the nasal mucosa. To determine the LXR expression patterns, the sinus mucosae and nasal mucosae obtained from patients with control and CRSsNP were collected, respectively, and IHC was performed using anti-LXRα or β Ab as a probe. In Figure 1, the LXRα was slightly distributed in the submucosal glands but largely expressed in the vessels of the controls. However, it was distributed in the epithelium (ep), vessels (v), glands (g) and stroma (st) of the CRSsNP group (panel A). In parallel, the LXRβ was mainly located at the submucosal glands and some stroma, both in the control and CRSsNP group (panel B).

**Figure 2:**
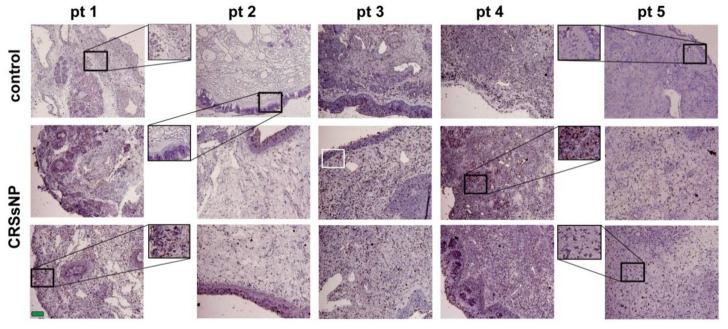
IHC analysis of lipid/fat deposition. The nasal mucosae were stained by Oil Red O and the dark reddish color spots indicate the presence of lipid/fat deposition. Some enlarged regions were 2 × magnified. Green scale bar = 100 μm.

Since cholesterol metabolites such as oxysterol are endogenous ligands for LXR [3], we next evaluated whether lipid/fat deposits in the human nasal mucosa. The oil red O was used as a dye for detecting lipid/fat deposition. In Figure 2, there were only some reddish spots, representing lipid/fat deposits, observed in the control specimens. However, more positive staining (dark reddish spots) was observed in the CRSsNP nasal mucosa (enlarged figures), suggesting that cholesterol and its metabolites may increasingly deposit in nasal mucosa of CRSsNP.

### 3.2. GW3965 Enhances PTX3 Protein and mRNA Expression

**Figure 3:**
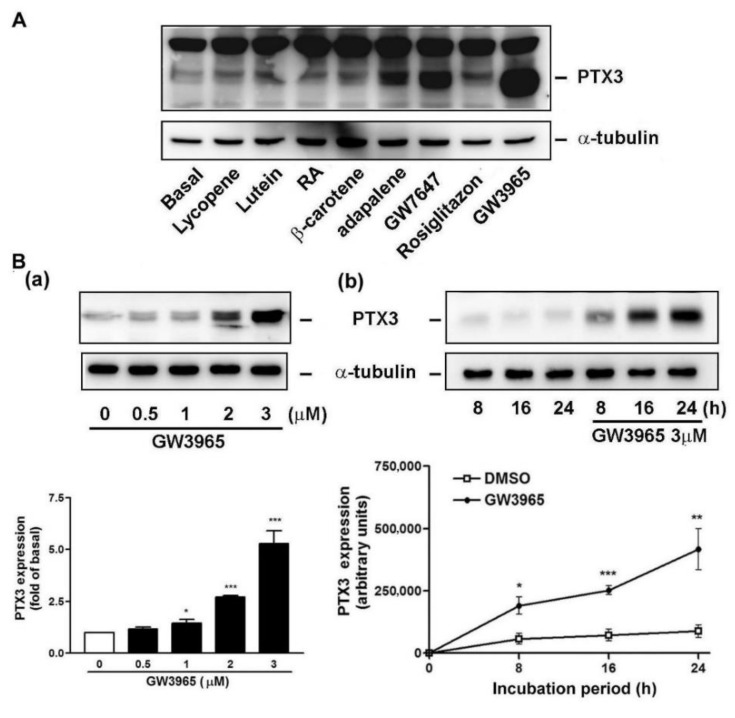
GW3965 induces PTX3 protein expression. (**A**) Effect of the nuclear receptor (NR) agonists on PTX3 expression. The fibroblasts were incubated with the NR-related compounds: carotenoids (lycopene, lutein, and β-carotene, 5 μM), retinoids (retinoic acid; RA, 1 μM and adapalene, RAR β/γ agonist, 2 μM), LXR agonist (GW3965, 5 μM), and PPAR agonists (GW7647, a PPARα agonist, 10 μM and rosiglitazone, PPARγ agonist, 30 μM). (**B**) GW3965 induced PTX3 protein expression. The fibroblasts were treated with (**a**) the GW3965 doses for 16 h or (**b**) GW3965 for the time periods and then analyzed by Western blot and densitometric analysis (*n* = 4). Pentraxin-3 (PTX-3), retinoic acid (RA), and dimethyl sulfoxide (DMSO). * *p* < 0.05, ** *p* < 0.01, and *** *p* < 0.001 versus control (DMSO).

We have shown that both of LXRs and lipids were expressed in the nasal mucosa, especially in the CRSsNP tissue. Since much submucosal stroma was positively stained for LXRs, the effects of the NR agonists on PTX3 expression in fibroblast cells were examined, which is based on our previous findings that fibroblast cells exist in the submucosal stroma regions of nasal mucosa [35]. In Figure 3A, some known NR agonists were selected, including carotenoids (lycopene, lutein, and β-carotene), all-trans retinoic acid (RA; a RAR agonist), adapalene (a RAR β/γ agonist), GW7647 (a PPARα agonist), rosiglitazone (a PPARγ agonist) and GW3965 (a dual inducer for LXRα and β). As previously reported [28,38], two close PTX3 proteins migrated at ~40 kDa. Among these tested substances, GW7647 and GW3965 were found to significantly cause PTX3 expression in the hNMDFs. GW3965, a representative LXR agonist, was used throughout this study.

In Figure 3B, GW3965 (GW) caused PTX3 protein induction in a dose-dependent fashion (panels a). Meanwhile, GW also time-dependently enhanced PTX3 protein expression, which was slightly increased at 8 h and markedly increased at 24 h of treatment, respectively. On the contrary, the α-tubulin expression remained unchanged (panels b). The induction of PTX3 was not due to cytotoxicity of GW since the viability assay excluded that possibility (Supplementary Materials Figure S1).

**Figure 4:**
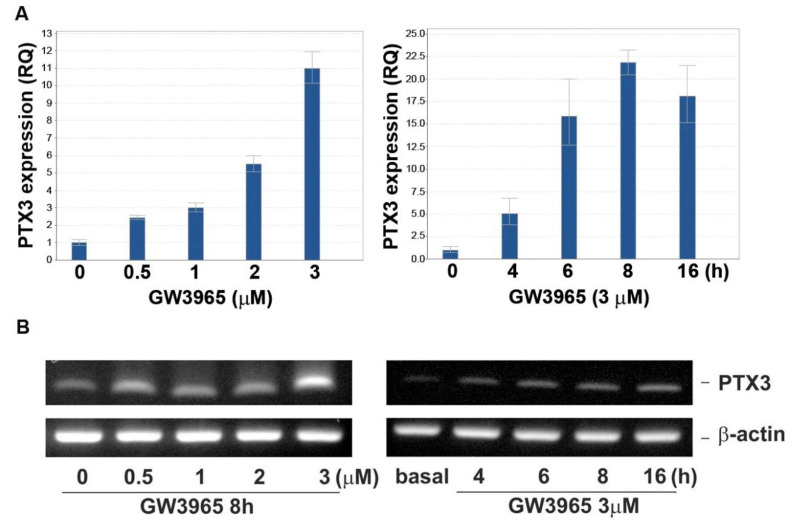
GW3965 induces ptx3 mRNA expression. The human fibroblasts were challenged with GW3965 for 8 h or GW3965 for the time periods. (**A**) Real-time PCR and (**B**) reverse transcription polymerase chain reaction (RT-PCR) were used to measure the ptx3 mRNA level (*n* = 3).

Next, the ptx3 mRNA expression level by GW challenge was explored. In Figure 4, GW3965 increased ptx3 mRNA level when the dose and time were increased, which reached a plateau at 8–16 h of treatment as assayed by the real-time PCR (panel A) and RT-PCR (panel B).

### 3.3. GW3965 Enhances PTX3 Protein Release

**Figure 5:**
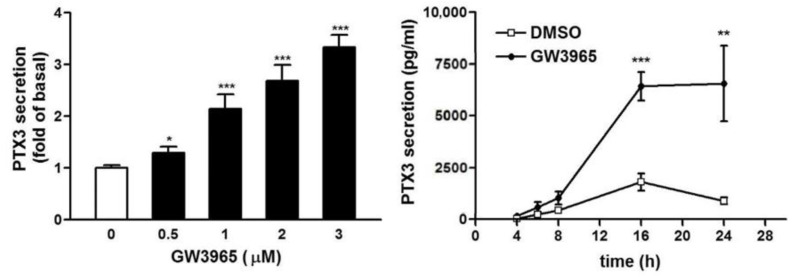
GW3965 induces PTX3 protein release in fibroblast culture medium. The human fibroblasts were treated with (left panel) the GW3965 for 16 h or (right panel) vehicle (DMSO) or GW3965 (3 μM). The PTX3 level in culture medium was measured by ELISA (*n* = 4). * *p* < 0.05, ** *p* < 0.01 and *** *p* < 0.001 versus control (0 μM of GW3965 or DMSO only).

Since the PTX3 protein expression was enhanced in the cells, whether PTX3 is releasable was examined by ELISA. In Figure 5, GW3965 treatment caused a substantial increase in PTX3 level and 0.5 μg/mL of GW could be significantly effective. In the meantime, the PTX3 secretion reached plateau at 16–24 h of GW treatment. The maximal PTX3 concentration was about 7000 pg/mL. This indicates that the produced PTX3 was released outside the cells and its concentration near nanomolar range could be functionally active in vivo.

### 3.4. An Endogenous LXR Agonist-Cholesterol Induces PTX3 Expression

**Figure 6:**
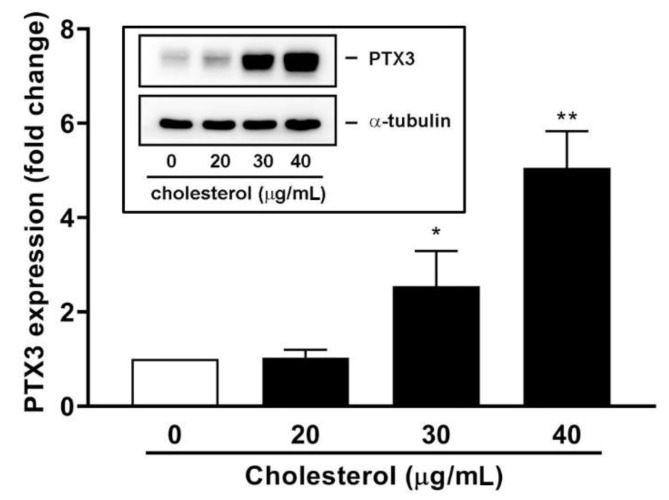
Effect of cholesterol on PTX3 protein expression. The fibroblasts were incubated with water-soluble cholesterol for 16 h. Western blot analysis and densitometry were performed to examine protein expression level (*n* = 4). Inset panel is a representative result of WB results. * *p* < 0.05 and ** *p* < 0.01 versus control (0 μg/mL).

The endogenous LXR agonist-cholesterol was used to verify the effects of GW3965 on PTX3 expression. Water-soluble cholesterol, which can lead to transdifferentiation of vascular smooth muscle cells (SMCs) into macrophage cells [39], was tested for its effect on PTX3 expression. As shown in Figure 6, cholesterol enhanced PTX3 protein expression level in hNMDFs and the inductory effect was significant at concentrations of 30 and 40 μg/mL.

### 3.5. Knockdown of LXRα and β Expression Compromises GW3965-Induced PTX3 Expression

**Figure 7:**
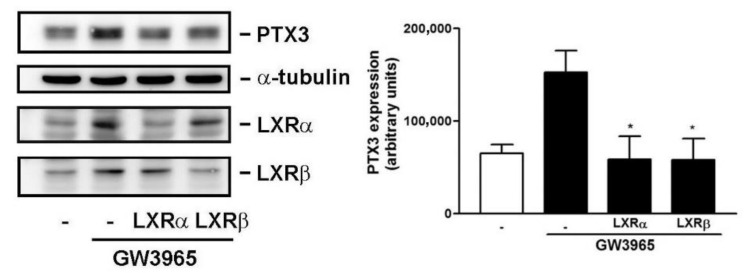
Effect of LXR siRNA interference on GW3965-induced PTX3 expression. Human fibroblasts were transfected with the indicated siRNA (-, control siRNA) and then treated with GW3965 (3 μM) for 16 h. Western blotting and densitometry were used to analyze the LXRα, LXRβ, PTX3 and α-tubulin expression (*n* = 4). * *p* < 0.05 versus control (control siRNA (-) and GW3965 treatment).

To confirm the importance of LXRα and β in GW3965-induced PTX3 expression, knockdown (KD) assay by siRNA interference was performed. The siRNA KD, indeed, reduced LXR α and β expression, respectively (Figure 7, left panel). Concomitantly, PTX3 expression was inhibited when LXR α and β expression were knocked down, respectively (right panel), suggesting that both LXR α and β are required for GW3965-induced PTX3 expression.

### 3.6. A Collaboration of PI3K/Akt and LXR Activation in PTX3 Induction

**Figure 8:**
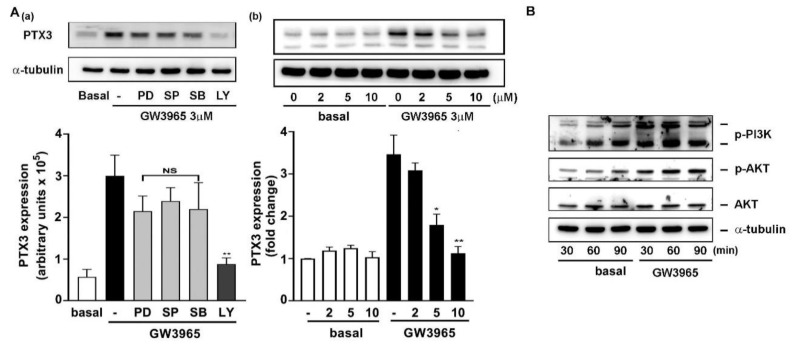
A collaboration of PI3K/Akt and LXR activation in PTX3 induction. (**A**) Effect of PI3K/Akt- and MAPK-related pathways on PTX3 induction. The fibroblasts were stimulated by vehicle (basal) or GW3965 with the inhibitors (10 μM each) or LY294002 (*n* = 3–4). (**B**) Effect of GW3965 on PI3K/Akt activation. The fibroblasts were incubated with vehicle (basal) or GW3965 (3 μM) for the time intervals (*n* = 3). Western blotting and densitometry were performed to assay protein level. NS: nonsignificant. PD098059 (PD), SP600125 (SP), SB202190 (SB), LY294002 (LY), protein kinase B (AKt). * *p* < 0.05 and ** *p* < 0.01 versus control.

Some studies have reported that the LPS response in macrophages can be promoted by LXR activation [40]. Next, we investigated whether GW3965-idncuced PTX3 expression involves cellular kinases and mitogen-activated protein kinases (MAPKs) signaling pathways. The pharmacological inhibitors targeting PI3K/Akt and MAPKs were used, including the inhibitor PD098058 (PD) for MAPKK, SP600125 (SP) for JNK1/2, SB202130 (SB) for p38 MAPK, and LY294002 (LY) for PI3K/Akt. Among these inhibitors, LY294002 for PI3K/Akt largely inhibited GW3965-induced PTX3 expression (Figure 8A, panel a). Moreover, the LY294002 attenuated GW-induced PTX3 expression when concentrations were increased (panel b). In parallel, GW treatment directly promoted PI3K and Akt phosphorylation (activation) (Figure 8B). Therefore, these results revealed that PI3K/Akt-related pathway also participates in GW3965-mediated PTX3 induction.

## 4. Discussion

Of the two types of CRS, CRSwNP accounts for 20–33% of cases, whereas CRSsNP accounts for the majority of CRS cases (~60%) [41]. Moreover, the LXR distributions in normal and CRSsNP sinonasal cavity have not been clearly determined. In this study, we were first to show that LXRα and β were most expressed in the mucosal glands and stroma in sinonasal mucosa of the CRSsNP patients. Interestingly, LXRα was found in the vascular endothelium of the control nasal mucosae, and both LXRα and β were expressed in the (submucosal) stroma. Based on our previous study, the stroma contains fibroblasts [35] and extracellular matrix proteins [42]. Therefore, it is reasonable that the CRSsNP-derived fibroblasts were used as the main material in this study. In addition, our previous study showed that the PTX3 protein is expressed in the submucosal stroma area [28]. Therefore, it is highly suspected that LXRs and PTX3 may be colocalized at this area.

In our screening, it was found that both the LXR and PPARα agonist, but not PPARγ, could induce PTX3 expression and release in hNMDFs (Figure 3A). Therefore, although it was recently reported that PPARγ agonists possess anti-inflammatory activity, PPARα agonists may also have a role in immunity and inflammation in CRSsNP, as reported in other systems [43]. Regarding whether lipids and fats are distributed within nasal mucosa, our results revealed that lipid/fat deposition was apparently increased in the CRSsNP sinonasal mucosa (Figure 2). Therefore, the lipid deposition and NR expression may play a certain role in immunity in sinonasal cavity in maintaining homeostasis during disease progression of CRS. Indeed, a literature search indicated that some studies have reported lipid deposition in the nasal cavity. For example, in guinea pigs, higher synthetic activities of cholesterol, and activity of cholesterol regulatory enzymes such as cholesterol sulfotransferase, are found in the nasal mucosae [44]. Moreover, human nasal fluid (NF) is rich in lipids (cholesteryl linoleate and arachidonate), contributing to antibacterial activity [45]. It was recently reported that nasal secretions of CRS patients appear to show increased levels of antimicrobial cholesteryl ester (CE) lipids [46] and an increased synthesis in antimicrobial CE production was observed in the nasal sinus tissue of CRSsNP patients [47]. These findings support our observations of an apparent increase in lipid/fat deposition in CRSsNP nasal mucosa (Figure 2) and of the roles of lipid/fat in antimicrobial activities. Our further results also showed that cholesterol was able to cause PTX3 expression at 30 and 40 μg/mL in an in-vitro cell model (Figure 6). The concentrations could be achieved since Lee and his colleagues have demonstrated that normal human NF contains all lipid types, including cholesterol, at about 40 μg/mL, reaching up to 90 μg/mL and CEs at about 25 μg/mL [45].

The PTX3 induced by LXR activation could be detected both in nasal fibroblasts and the culture medium (Figure 5). Numerous studies have noted bacteria in the pathogenesis of CRS [24,25]. Our previous study showed that PTX3 was upregulated in CRSsNP compared with controls [28]. Therefore, the ability of LXR activation to increase of PTX3 and stromal fibroblasts are highly suspected to be involved in CRSsNP pathogenesis and pathophysiology. Since one of the most important roles for pentraxins is against bacteria [48], the overexpressed PTX3 by LXR in nasal mucosa could contribute to defense of bacterial infections. However, it was found that the ptx3 transcript and protein was not triggered at the early stage (<4 h) of GW treatment (Figure 3 and Figure 4). Therefore, it seems that LXR activation is not an early responder for PTX3 induction during CRSsNP progression.

In the current study, our results suggested PTX3 expression mainly through LXRα and β activation (Figure 7). A previous study showed that Pu-1, AP1, NF-κB, SP1 and NF-IL6 binding sequences are located at the promoter region of human ptx3 gene [49], and NF-κB and CREB are needed for peptidoglycan-mediated PTX3 production [28]. Intriguingly, a binding site analysis showed that there is no LXR binding site in the 1317-bp promotor region of human ptx3 gene. It is possible that the binding sites for LXRs don’t locate within the analyzed region, or LXR can bind to some unidentified binding sequences. Nevertheless, some binding sites for the LXR heterodimer, including RXR, SXR: RXR and PPAR: RXR, could be found in the promoter region. Moreover, a collaboration of PI3K/Akt with LXR activation is involved in this induction (Figure 8). A previous study has shown that JNK and PI3K participate in the LXR-mediated induction of gene expression in macrophages [50] and the LXR agonist GW3965 induces Akt phosphorylation in cultured neural progenitor cells [51]. The PI3K/Akt signaling pathway may follow LXR activation or act independently to mediate GW3965-induced PTX3 expression. The induction of noncanonical pathways by NR agonism has also been observed in steroidal estrogen [52], vitamin D3 [53] and RAR and RXR agonists such as retinol and RA in promoting MMP-2 activity by different signaling pathways in Sertoli cells through inducing ERK1/2 phosphorylation [54]. Accordingly, how LXRs cooperate with PI3K/Akt needs to be further investigated.

## 5. Conclusions

We provide here the first evidence that LXR activation by a selective LXR agonist-GW3965 and cholesterol induces long PTX3 expression and release in hNMDFs through activation of LXRα and β, and involvement of the PI3K/Akt signaling pathway. We also provide the first evidence showing that the expression of LXRα and β and deposition of lipid/fat are increased in CRSsNP nasal mucosa. The study demonstrates the possible cooperative role of lipids and LXRs in regulating pentraxin 3 expression, which may participate in microbial recognition and clearance for defense against invasive pathogens and tissue repair. The LXR agonist may be further developed in preventing CRSsNP development and progression.

## Figures and Tables

**Table 1:** Primers sets for real-time- and RT-PCR.

Gene	Forward Primer (5′-3′)	Reverse Primer (5′-3′)	Product Size (bp)
ptx3	GCTCTCTGGTCTGCAGTGTT	CTTGTCCCATTCCGAGTGCT	147
β-actin	ATCATGTTTGAGACCTTCAA	CATCTCTTGCTCGAAGTCCA	314
Cyc-A	TATCTGCACTGCCAAGACTGAGTG	CTTCTTGCTGGTCTTGCCATTCC	127

## Data Availability

The data used to support the findings of this study will be provided upon request by the Journal.

## Supplementary Materials

The following are available online at https://www.mdpi.com/2077-0383/10/3/452/s1, Figure S1: Effect of GW3965 on viability of hNMDFs.
