# Supplementary material for: Liver X Receptor Expression and Pentraxin 3 Production in Chronic Rhinosinusitis and Sinonasal Mucosal Fibroblast Cells
Source: J Clin Med. 2021 Jan 25;10(3):452. doi: 10.3390/jcm10030452 (PMC7865759; doi:10.3390/jcm10030452)
Supplement: Supplementary file 1 [file jcm-10-00452-s001.pdf]

Supplementary data
Materials and Methods

Materials

3-(4,5-dimethylthiazol-2-yl)-2,5-diphenyl-2H-tetrazolium bromide (MTT) was purchased from Sigma-Aldrich Chemical Co. (St Louis, MO, USA).

Cell viability assay (MTT assay)

Cells growing in complete medium and reaching 80-90% of confluency were treated with GW3965 for the indicated times. After incubation, cells were added with 0.5mg/ml MTT for 2 h at 37°C. Formazan crystals resulting from MTT reduction were dissolved by adding DMSO and gently agitating for 20 min. The absorbance of the supernatant was then measured spectrophotometrically in an ELISA reader at 550 nm.

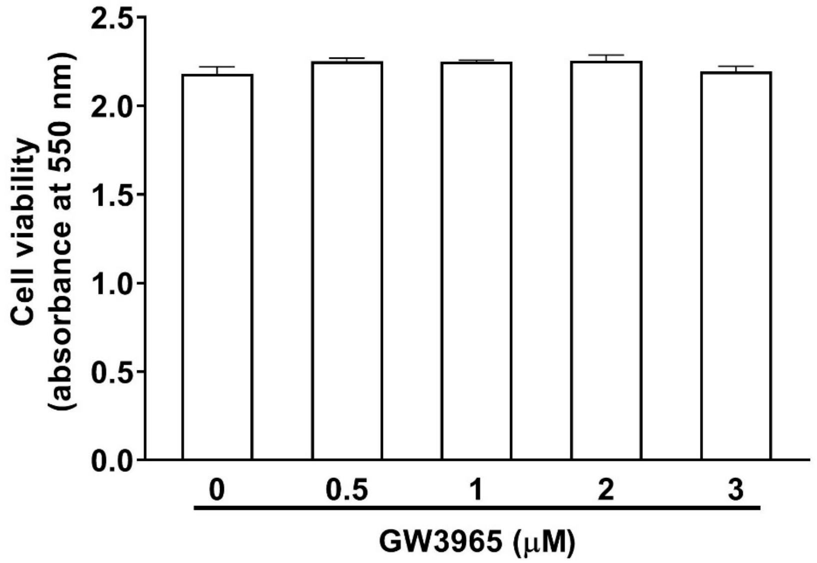

Figure S1. Effect of GW3965 on viability of hNMDFs. Cells were treated with GW3965 for 16 h. After the treatment, cell viability was determined by MTT assay. This is representative from four similar experiments.
